# Cutaneous Crohn’s disease unmasked during interleukin 17 inhibition for hidradenitis suppurativa: A 2-patient case series

**DOI:** 10.1016/j.jdcr.2026.06.041

**Published:** 2026-06-26

**Authors:** Ysaac Zegeye, Saranya Ravi, Carrie Diamond, Ashley Rose Scholl Loew, Maria Angelica Selim, Anne L. Marano, Amber Fresco

**Affiliations:** a Duke University School of Medicine, Durham, North Carolina; b Department of Dermatology, Duke University Medical Center, Durham, North Carolina; c Department of Pathology, Duke University Health System, Durham, North Carolina

**Keywords:** cutaneous Crohn’s disease, granulomatous inflammation, hidradenitis suppurativa, interleukin 17 inhibitor

## Case reports

Hidradenitis suppurativa (HS) is a chronic, debilitating condition of the hair and apocrine gland-bearing intertriginous areas; it is characterized by recurrent, painful abscesses, nodules, and sinus tracts.^1^ Treatment with systemic biologic therapies has become a critical asset in the management of HS.^2^ However, a subset of patients endure refractory disease or adverse effects. Interleukin (IL) 17 inhibitors, such as secukinumab and bimekizumab, are emerging therapeutics that target proinflammatory cytokine T helper 17–driven pathways within HS pathogenesis. Despite IL-17’s role in intestinal barrier maintenance, IL-17 inhibitors have been associated with new onset and exacerbation of inflammatory bowel disease (IBD).^3^ These cases highlight the potential for IL-17 inhibition to unmask or exacerbate cutaneous Crohn’s disease in HS and emphasize the importance of gastrointestinal screening prior to treatment.

### Case 1

A 52-year-old woman with HS, Hurley stage III, presented for management of severe disease involving the groin and intertriginous areas. She had failed multiple therapies, including adalimumab and infliximab. After an insurance change, she transitioned to secukinumab 300 mg monthly following a single loading dose of bimekizumab 320 mg. Secukinumab initially improved tenderness and drainage; however, 2 months after initiation, physical examination revealed linear fissures of the gluteal fold and groin with erythema and vulvar edema (Fig 1). She additionally reported rectal bleeding and abdominal pain, which previously was attributed to hemorrhoids confirmed on colonoscopy 3 years prior.

**Fig 1:**
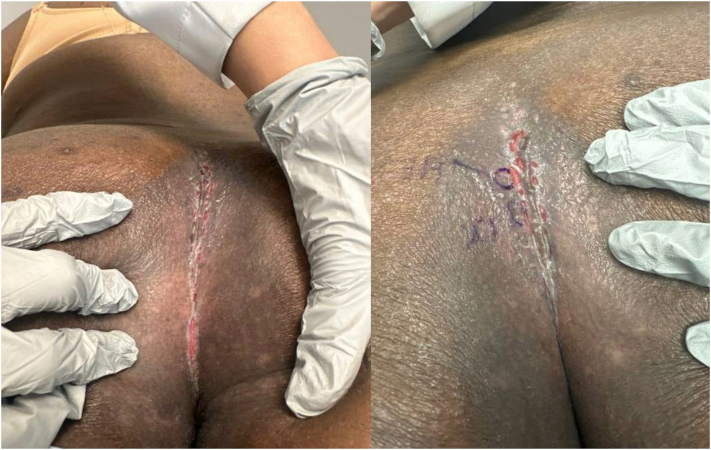
Physical examination findings after initiation of secukinumab. Linear, erythematous ulceration with cribriform appearance extending vertically along the midline gluteal cleft with additional short linear areas of ulceration adjacent to the primary fissure.

Punch biopsy of the gluteal fold revealed epidermal acanthosis with mild spongiosis and a dense perivascular and periadnexal lymphoplasmacytic infiltrate containing multinucleated giant cells, as well as well-formed, nonnecrotizing granulomas throughout the mid dermis (Fig 2, *A* and *B*). The patient’s cutaneous presentation and gastrointestinal symptoms raised suspicion of cutaneous Crohn’s disease.

**Fig 2:**
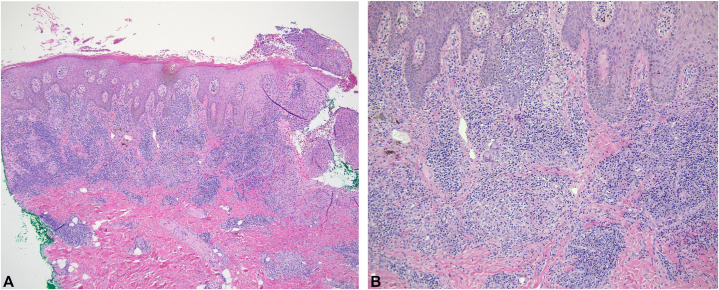
Histology from punch biopsy of linear ulceration on gluteal cleft. **A,** Acanthosis, mild spongiosis, and a superficial to middermal perivascular and periadnexal lymphoplasmacytic infiltrate with scattered neutrophils. **B,** Numerous multinucleated giant cells and nonnecrotizing granulomas are present throughout the papillary dermis. (**A** and **B,** Hematoxylin-eosin stain; original magnifications: **A,** ×40; **B,** ×100.)

Given the temporal relationship between IL-17 blockade and symptom onset, secukinumab was discontinued. Colonoscopy revealed localized erythema in the rectum, with biopsy of the right colon showing a rare superficial nonnecrotizing granuloma. Computed tomography (CT) enterography demonstrated no definitive Crohn’s disease. After multidisciplinary discussion, despite the minimal luminal disease, the presentation of knife-like ulcerations and nonnecrotizing granulomatous histology was considered most consistent with cutaneous Crohn’s disease, likely unmasked by IL-17 inhibition.

Ustekinumab 90 mg every 8 weeks was initiated for treatment of both HS and Crohn’s disease. The patient initially reported reduced pain but discontinued therapy due to severe headaches. She was then transitioned to guselkumab 100 mg every 8 weeks and showed significant improvement with minimal inflammatory activity after 2 months.

### Case 2

A 41-year-old woman with HS, Hurley stage III, presented to the emergency department with 6 weeks of rapidly progressive painful lesions involving the buttocks, axillae, and trunk. She was diagnosed with HS in 2019 and had failed numerous biologics, most recently infliximab. She had recently started secukinumab and received 2 loading doses.

In the weeks preceding and following initiation of secukinumab, new ulcerative plaques developed in the patient. She also reported hematochezia with intermittent bloating. Examination revealed numerous brightly erythematous plaques of the thighs, abdomen, buttocks, and extremities with gunmetal gray borders and cribriform scarring, as well as friable tissue and exudate (Fig 3). Given her severity, she was admitted for further diagnostic investigation and initiation of systemic therapy.

**Fig 3:**
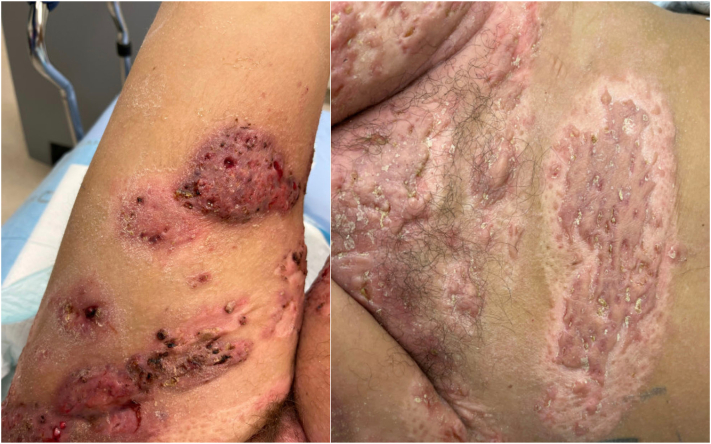
Physical examination findings of the upper portion of the left thigh and lower portion of the abdomen demonstrating brightly erythematous plaques with gunmetal gray borders and cribriform scarring, as well as admixed bleeding, friable tissue, and exudate.

A punch biopsy of the upper portion of the left thigh demonstrated neutrophilic collections extending into the subcutaneous tissue and ill-defined, nonnecrotizing granulomas (Fig 4, *A* and *B*). The differential diagnosis included pyoderma gangrenosum, cutaneous Crohn’s disease, and sarcoidosis.

**Fig 4:**
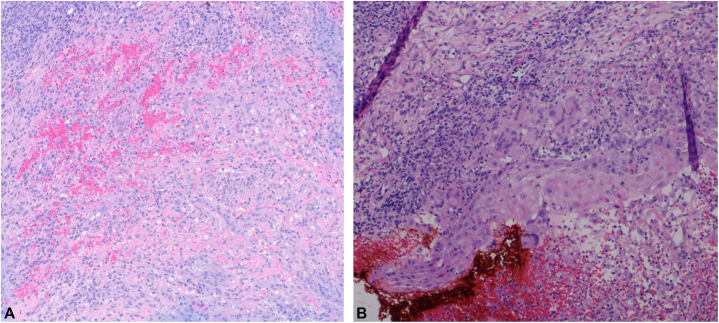
Histology from punch biopsy of the upper portion of the left thigh. **A,** Collections of histiocytes and nonnecrotizing granulomas in the dermis. **B,** Nonnecrotizing granulomatous inflammation with neutrophil collections and scattered plasma cells. (**A** and **B,** Hematoxylin-eosin stain; original magnifications: **A,** ×100; **B,** ×100.)

Colonoscopy demonstrated gastric erosions without mucosal evidence of Crohn’s disease, while CT enterography revealed a 7-cm segment of nonspecific small bowel inflammation. Colonic biopsies were unrevealing.

Given the constellation of findings with gastrointestinal symptoms, new cutaneous lesions, small bowel inflammation, and histopathologic findings in the setting of IL-17 inhibition, there was concern for either IBD-spectrum or neutrophilic inflammatory process. Gastroenterology evaluation raised concern for Crohn’s-spectrum disease based on the clinical, pathologic, and imaging findings, although this was not definitive. Although the histopathologic findings were nonspecific and may overlap with HS, they informed clinical decision making by prompting multidisciplinary evaluation and discontinuation of secukinumab. The patient was treated with systemic corticosteroids, cyclosporine, and initiated on ustekinumab-ttwe (390 mg loading dose followed by 90 mg maintenance every 8 weeks), resulting in clinical stabilization.

## Discussion

HS and IBD share inflammatory pathways that involve dysregulation of tumor necrosis factor-α and IL-23/IL-17 axes. Notably, patients with HS exhibit a higher baseline prevalence of Crohn’s disease than the general population, supporting a shared inflammatory predisposition.^4^ IL-17 plays a key role in antibacterial defense and intestinal epithelial repair; however, inhibition may impair mucosal homeostasis and promote dysregulated T helper 1/IL-23–mediated inflammation in genetically predisposed individuals. Although IL-17 inhibitors are effective in HS, new onset or exacerbation of IBD has been observed.^3^

While IL-17 inhibitor–associated IBD is well established, association with cutaneous Crohn’s disease and granulomatous skin manifestations remain poorly defined. In case 1, the onset of fissures during IL-17 inhibition with biopsy-proven granulomatous dermatitis raised concern for Crohn’s disease. Although colonoscopy and CT imaging lacked characteristic findings, cutaneous Crohn’s disease may precede or manifest independently of intestinal involvement.^5^ Similarly, case 2 demonstrated cutaneous granulomatous inflammation and small bowel changes in the setting of IL-17 inhibition without fully meeting the criteria for Crohn’s disease. Together, these cases demonstrate that cutaneous inflammation may emerge without definitive endoscopic disease. However, granulomatous inflammation alone may not reliably distinguish HS from cutaneous Crohn’s disease.^6^^,^^7^ In both cases, management decisions were guided by clinicopathologic correlation rather than histopathology alone. Discontinuation of secukinumab, adjunctive immunosuppression, and transition to alternative biologics resulted in clinical improvement of skin and gastrointestinal symptoms.

These cases highlight a spectrum of inflammatory presentations in the setting of IL-17 inhibition, ranging from biopsy-supported cutaneous Crohn’s disease to less specific granulomatous inflammatory processes. Additionally, they underscore the importance of screening for IBD symptoms before initiating IL-17 inhibitors in HS. While these cases do not establish causality, they add to the growing evidence that IL-17 inhibition may precipitate Crohn’s disease, including cutaneous Crohn’s disease in predisposed individuals. Clinicians should maintain suspicion for cutaneous Crohn’s disease in patients with HS in whom fissures and skin ulcers develop, even in the absence of gastrointestinal symptoms or colonic involvement, particularly in the context of IL-17 blockade. Cessation of offending IL-17 inhibitor, multidisciplinary collaboration, and biologic redirection are essential in preventing morbidity.

## Conflicts of interest

Dr Marano serves as a consultant for Immunovant and as a principal investigator for studies sponsored by Biogen, Bristol Myers Squibb, Priovant, Immunovant, and Merck and has previously served as an investigator for Viela Bio. Dr Fresco serves as a subinvestigator for UCB Biopharma. Drs Ravi, Diamond, Loew, and Selim and author Zegeye have no conflicts of interest to declare.
